# Veterinary World reviewer acknowledgment 2018

**DOI:** 10.14202/vetworld.2019.85-89

**Published:** 2019-01-16

**Authors:** A. V. Sherasiya, Nazir

**Affiliations:** Veterinary World, Star, Gulshan Park, NH-8A, Chandrapur Road, Wankaner – 363621, Dist. Morbi, Gujarat, India

**Contributing reviewers**

Veterinary World editorial team would sincerely like to thank all of our reviewers who contributed to peer review for the journal in 2018.

**A. Aygun** Turkey

**A. I. Pastiu** Romania

**A. R. Silva** Brazil

**Abdelaziz ED-DRA** Morocco

**Abdelkbir Rhalem** Morocco

**Abdullatif. A. Asheg** Libya

**Abhishek Tripathi** USA

**Adamu Saleh Saidu** Nigeria

**Adriana Titilincu** Romania

**Ahmad Daryani** Iran

**Ahmed S. Abdoon** Egypt

**Ailian Geng** China

**Alberto Elmi** Italy

**Alejandro Hidalgo** Chile

**Alessandra Pelagalli** Italy

**Alessandra Torina** Mexico

**Amany Adel** Egypt

**Amir Mohammad Mortazavian** Iran

**Amira Taman** Egypt

**Amitav Bhattacharya** India

**Amitava Dey** India

**Amlan K. Patra** India

**Amy C. Murillo** USA

**Anand SP** India

**Andres Carvajal** Chile

**Angelina Bossi Fraga** Brazil

**Anil Kumar Arora** India

**Antonella Lo Nostro** Italy

**Antoni Prenafeta** Spain

**Antonio Bevilacqua** Italy

**Anut Chantiratikul** Thailand

**Arathy D. S. Nair** USA

**Archana Jain** India

**Archana Verma** India

**Arda Sozcu** Turkey

**Arda Yildirim** Turkey

**Arya Sobhakumari** USA

**Ashish Chopra** India

**Ashok K. Chockalingam** USA

**Ashraf M. Abu-Seida** Egypt

**Ashutosh Wadhwa** USA

**Ashwani Kumar** India

**Ashwell Ndhlala** South Africa

**Ayman Abdel-Aziz Swelum** Egypt

**B. Hoffmann** Germany

**B. D. Enger** USA

**Bala Manickam** USA

**Balamurugan Vinayagamurthy** India

**Bambang Pontjo Priosoeryanto** Indonesia

**Bangyuan Wu** China

**Baratang Alison Lubisi** South Africa

**Bartosz Kieronczyk** Poland

**Belgin Siriken** Turkey

**BKKK Jinadasa** Sri Lanka

**Breda Jakovac-Strajn** Slovenia

**Buket Er Demirhan** Turkey

**Carlos A. Rossetti** Argentina

**Chandan Paswan** India

**Chandramani Pathak** India

**Charleata A. Carter** USA

**Chen Yao** USA

**Cheng-Hung LAI** Taiwan

**Christian Tiambo Keambou** Kenya

**Chrysostomos Vasilios Milis** Greece

**Chyer Kim** USA

**Claudia Bezerra da Silva** Brazil

**Daniel M. Aguiar** Brazil

**David Gleeson** Ireland

**Deepmala Agarwal** USA

**Deepti Chachra** India

**Dinh Ngoc Nguyen** Australia

**Duane L. Garner Garner** USA

**Eduardo Jorge Boeri** Argentina

**Edward Ferguson** USA

**Ehsan Mostafavi** Iran

**Ekene Vivienne Ezenduka** Nigeria

**El Amiri Bouchra El Amiri** Morocco

**Elif Çelik** Turkey

**Elina B. Reinoso** Argentina

**Eloiza Teles Caldart** Brazil

**Eva Voslarova Czech** epublic

**Ewaldus Wera** Indonesia

**Filippo Fratini Fratini** Italy

**Filippo Giarratana** Italy

**Florence D. Tarfa** Nigeria

**Fouad Kasim Mohammad** Iraq

**Genevieve Andre-Fontaine** France

**Gnanavel Venkatesan** India

**Guilherme Dias de Melo** France

**Gul Ahmad** USA

**Guoxiang Chao** China

**Guo-Zhong Zhang** China

**Gyanendra Gongal** India

**H.T Chen** China

**Hamed Kalani** Iran

**Hanna Markiewicz** Poland

**Hany Mhany Ragab Abdel-Latif** Egypt

**Hasan Meydan** Turkey

**Ho Seong Seo** Korea

**Hope Otsyina Otsyina** Ghana

**Hudson A. Pinto** Brazil

**Huiling Sun** China

**Iddya Karunasagar** India

**Idrees Mehraj Allaie** India

**Imen Belhadj Slimen** Tunisia

**Inga Stadaliene** Lithuania

**Ionel D. Bondoc** Romania

**Ismail Hakki Tekiner** Turkey

**Jamal Gharekhani** Iran

**Jana Jankovicová** Slovakia

**Jean-Mathieu Bart** France

**Jerome Andonissamy** India

**Joao Simoes** Portugal

**João Simões** Portugal

**John White** Australia

**Josef Illek Czech** Republic

**Juan Morgaz** Spain

**Kai Huang** USA

**Kamal Niaz** Pakistan

**Kameshwar P. Singh** USA

**Kannan Thandavan Arthanari** India

**Karim El-Sabrout** Egypt

**Kavous Solhjoo** Iran

**Kekungu Puro** India

**Kemin Xu** USA

**Kiril M. Dimitrov** USA

**Koushlesh Ranjan** India

**Kumar Venkitanarayanan** USA

**Kumari Sunita** India

**L. C. Penning** Netherland

**Laura Bongiovanni** Italy

**Laxmi Narayan Sarangi** India

**Lenin Lenin Aguirre Riofrio** Ecuador

**Liben Chen** USA

**Liliana Aguilar-Marcelino** Mexico

**Linous Munsimbwe** Zambia

**Luis Souza Lima de Souza Reis** Brazil

**Luiz Otavio Pereira Carvalho** Brazil

**M. Samardžija** Croatia

**Mahmoud Mohey Elhaig** Egypt

**Maja Josip Velhner** Serbia

**Mallikarjun Bidarimath** USA

**Maria Emilia Eirin** Argentina

**Mario Manuel Dinis Ginja** Portugal

**Marta Vascellari** Italy

**Maryam Azizkhani** Iran

**Mathias Devreese** Belgium

**Md. Fakruddin** Bangladesh

**Md.Tanvir Rahman** Bangladesh

**Mehmet Fatih Aydin** Turkey

**Mette B. Petersen** Denmark

**Miguel Quaresma** Portugal

**Minakshi Prasad** India

**Miroslaw Mariusz Michalski** Poland

**Mohamed Haroun** Qatar

**Mohammed El-Houadfi** Morocco

**Mohsen Najimi** Iran

**Mramba Nyindo Nyindo** Tanzania

**Muhammad Irfan-ur-Rehman Khan** Pakistan

**Muhammad Naeem Iqbal** China

**Münir Aktas** Turkey

**N. De Briyne** Belgium

**N. Punniamurthy**, Natesan India

**Nadeem Shabir** India

**Naim Deniz Ayaz** Turkey

**Nares Trakooljul** Germany

**Nazli Ercan** Turkey

**Nicholas Johnson** UK

**Nicole Borel** Switzerland

**Nitesh Kumar** India

**Noha Salem** Egypt

**Nuh Kiliç** Turkey

**Olfat Anter Mahdy** Egypt

**Oludayo O. Fashina** South Africa

**Osama Hassan Omer** Sudan

**Osman Yasar TeL** Turkey

**Ovais Aarif** India

**P. L Toutain** France

**Pabitra Hriday Patra** UK

**Panagiotis E. Simitzis** Greece

**Panch Kishor Bharti** India

**Parag Nigam** India

**Partha Ray** UK

**Patrick Hensel** Switzerland

**Pinar Peker Akalin** Turkey

**Pingli He** China

**Pourouchottamane.R** India

**Prof. Aulanniam** Indonesia

**R. Arora** India

**R. Somvanshi** India

**R. C. Stocco** Brazil

**Rajani Ravindranath Thanissery** USA

**Ramgopal Laha** India

**Ramute Miseikiene** Lithuania

**Ranganath Mamidi** USA

**Raquel Salazar Lugo** Venezuela

**Rasheed Adetola Ajadi** Nigeria

**Roberto Condoleo** Italy

**Rodrigo Alberto Jerez Ebensperger** Spain

**Roselene Ecco** Brazil

**Ruben Pérez** Uruguay

**Rute M. Noiva** Portugal

**S. Vince** Croatia

**Sadia Benamrouz** France

**Sana A. M. Elgayar** Egypt

**Sandeep Bhatia** India

**Sandeep Ghatak** India

**Santanu Banik** India

**Saravanan Ramakrishnan** India

**Sevda Guzel** Turkey

**Shahzad Ali** Pakistan

**Shailbala Singh** USA

**Shambhunath Choudhary** USA

**Shawky M. Aboelhadid** Egypt

**Shumaila Arif** Australia

**Sibele Borsuk** Brazil

**Simona Martinotti** Italy

**Sinan Ince** Turkey

**Socorro Retana-Marquez** Mexico

**Sreenu Makkena** India

**Stefania Perrucci** Italy

**Stefano D’Amelio** Italy

**Süleyman çilek** Turkey

**Surasakdi Wongratanacheewin** Thailand

**Suresh H. Basagoudanavar** India

**Takumi Shinkai** Japan

**Tanko Polycarp Nwunuji** Nigeria

**Tapas Kumar Sar** India

**Thomas J. Nolan** USA

**Tomas Erban Czech** Republic

**Tsutomu Sekizaki** Japan

**V. C. Rayulu** India

**Vahid Noaman** Iran

**Varij Nayan** India

**Vassilis Papatsiros** Greece

**Vikas Vohra** India

**Vishal S. Suthar** India

**Visnuvinayagam Sivam** India

**Walied Abdo** Egypt

**Widya Paramita Lokapirnasari** Indonesia

**Xiaonan Yang** China

**Xu Gu** China

**Ya-Ching Lin** Taiwan

**Yahya Tahamtan** Iran

**Ya-Ling Huang** Taiwan

**Yan-Chen Bo** China

**Yassir Adam Shuaib** Sudan

**Yuke Wang** USA

**Yves Millemann** France

**Zhuo-Ming Qin** China

**Zuhair Ismail** Jordan

